# Distinct mechanisms of Up state maintenance in the medial entorhinal cortex and neocortex

**DOI:** 10.1016/j.neuropharm.2016.11.009

**Published:** 2017-02

**Authors:** Richard J. Digby, Diego S. Bravo, Ole Paulsen, Vincent Magloire

**Affiliations:** a Department of Physiology, Development and Neuroscience, Physiological Laboratory, University of Cambridge, Downing Street, Cambridge, CB2 3EG, UK; b Department of Clinical and Experimental Epilepsy, Institute of Neurology, University of College London, Queen Square, London, WC1N 3BG, UK

**Keywords:** Up state, Slow oscillation, Medial entorhinal cortex, Barrel cortex, Kainate receptor, NMDA receptor

## Abstract

The medial entorhinal cortex (mEC) is a key structure which controls the communication between the hippocampus and the neocortex. During slow-wave sleep, it stands out from other cortical regions by exhibiting persistent activity that outlasts neocortical Up states, decoupling the entorhinal cortex-hippocampal interaction from the neocortex. Here, we compared the mechanisms involved in the maintenance of the Up state in the barrel cortex (BC) and mEC using whole cell recordings in acute mouse brain slices. Bath application of an NMDA receptor antagonist abolished Up states in the BC, and reduced the incidence but not the duration of Up states in the mEC. Conversely, blockade of kainate receptors decreased Up state duration in the mEC, but not in the BC. Voltage clamp recordings demonstrated the presence of a non-NMDA glutamate receptor-mediated slow excitatory postsynaptic current, sensitive to the selective kainate receptor antagonist UBP-302, in layer III neurons of the mEC, which was not observed in the BC. Moreover, we found that kainate receptor-mediated currents assist in recovery back to the Up state membrane potential following a current-induced hyperpolarisation of individual cells in the mEC. Finally, we were able to generate Up state activity in a network model of exponential integrate-and-fire neurons only supported by AMPA and kainate receptor-mediated currents. We propose that synaptic kainate receptors are responsible for the unique properties of mEC Up states.

## Highlights

• mEC exhibits persistent Up state activity in the absence of neocortical input *in vitro.*
• NMDA receptors are required for Up states in the neocortex, but not in the mEC.
• Kainate receptors maintain Up states in the mEC.
• Kainate receptor currents are sufficient to maintain Up states *in silico*.

## 1 Introduction

The cortical slow oscillation is believed to be important for memory consolidation (Major and Tank, 2004, Compte, 2006, Dupret and Csicsvari, 2012). It consists of periods of intense synaptic activity from both excitatory and inhibitory neurons, known as Up states, separated by relatively quiescent Down states (Steriade et al., 1993a). Fluctuations between Up and Down states are seen *in vivo* during slow-wave sleep, quiet wakefulness, and anaesthesia (Steriade et al., 1993a, Hahn et al., 2012), and have also been recorded in different *ex vivo* preparations, including acute brain slices from various cortical areas, such as the prefrontal, barrel and medial entorhinal cortices (Sanchez-Vives and McCormick, 2000, Mann et al., 2009; Wester and Contreras, 2012).

Up states are generated intrinsically in the cortex and driven by glutamatergic synaptic transmission. Indeed, both α-amino-3-hydroxy-5-methyl-4-isoxazolepropionic acid (AMPA) and *N*-methyl-d-aspartate (NMDA) receptors are required for the generation of Up states in the neocortex (Sanchez-Vives and McCormick, 2000, Shu et al., 2003, Favero and Castro-Alamancos, 2013, Castro-Alamancos and Favero, 2015). Inhibitory synaptic transmission via type A gamma-aminobutyric acid (GABA_A_) receptors balances the excitation during Up states (Shu et al., 2003, Mann et al., 2009), while GABA_B_ receptors contribute to the Up-to-Down state transition (Mann et al., 2009, Craig et al., 2013).

The medial entorhinal cortex (mEC) is particularly interesting for the possible functions of Up-Down states in memory since it is the main cortical input and output of the hippocampus (Van Strien et al., 2009, Insausti, 1993), a structure essential for episodic memory (Squire, 1992). Interestingly, persistent network activity in the mEC outlasts neocortical Up states (Hahn et al., 2012) and the mEC could therefore act as an independent rhythm generator for slow oscillations. While neocortical Up states are initiated in layer V (Sanchez-Vives and McCormick, 2000, Beltramo et al., 2013, Stroh et al., 2013), mEC Up states are thought to be generated in the superficial layers (Cunningham et al., 2006, Tahvildari et al., 2012). In addition, only fast spiking interneurons seem to be involved in mEC Up states, while several other interneuron subtypes are firing during neocortical Up states (Neske et al., 2015). Interestingly, kainate receptors, a family of non-NMDA glutamate receptors, have been implicated in Up state activity in the mEC (Cunningham et al., 2006), and kainate receptor-mediated postsynaptic currents are present in neurons from the superficial layers (West et al., 2007).

However, the mechanism responsible for the maintenance of persistent mEC Up states, capable of skipping several cycles of neocortical Down state transitions, remains elusive. To investigate this question, we compared Up state activity in acute brain slices from the BC and the mEC, using whole-cell patch clamp recordings. Spontaneous Up states lasted longer in the mEC compared to the BC, suggesting different intrinsic mechanisms. Pharmacological blockade of NMDA or kainate receptors, combined with voltage clamp recordings, revealed that the difference in Up state duration between BC and mEC arises from a predominant contribution of kainate receptor-mediated currents to Up state activity in the mEC, in addition to the AMPA and NMDA receptor-mediated currents that drive neocortical Up states.

## 2 Materials and methods

### 2.1 Slice preparation

Wild type C57BL/6 mice of either sex (P14–P19, Harlan, Bicester, UK) were decapitated under deep isoflurane-induced anaesthesia under personal and project licenses held by the authors under British Home Office regulations. Young animals were used, consistent with many other *in vitro* studies, in order to ensure robust Up state activity, of an adequate frequency, in control conditions for experimental work and analysis (Mann et al., 2009, Tahvildari et al., 2012, Craig et al., 2013, Žiburkus et al., 2013). Horizontal mEC and coronal BC slices (400 μm) were prepared in ice cold (<4 °C) artificial cerebrospinal fluid (aCSF) using a vibrating blade microtome (Leica Biosystems, VT1200S, Germany) as previously described (Mann et al., 2009, Rodríguez-Moreno et al., 2013). Slices were then incubated for at least 90 min at room temperature (22–24 °C) in an interface chamber between aCSF and humidified carbogen gas consisting of 95% O_2_ and 5% CO_2_. Different aCSF compositions were used for Up state experiments and synaptic current recordings; however, the same aCSF composition was used for both mEC and BC slices and for both slicing and recording within each experiment. Experiments studying spontaneous and evoked Up states hence used aCSF composed of 126 mM NaCl, 3.5 mM KCl, 1.25 mM NaH_2_PO_4_, 1.2 mM CaCl_2_, 1 mM MgSO_4_, 26 mM NaHCO_3_, and 10 mM glucose (pH 7.2–7.4), while experiments investigating synaptic currents used aCSF composed of 126 mM NaCl, 3 mM KCl, 1.25 mM NaH_2_PO_4_, 2 mM CaCl_2_, 2 mM MgSO_4_, 26 mM NaHCO_3_, and 10 mM glucose (pH 7.2–7.4). Prior to recording, slices were mounted on coverslips coated with 0.1% poly-l-lysine in ultrapure H_2_O, and then transferred to a recording chamber in which aCSF was superfused at 4–5 ml/min and maintained at 30–32 °C. This method helps to preserve spontaneous activity in the slice (Hájos et al., 2009, Mann et al., 2009).

### 2.2 Electrophysiology

Whole-cell patch clamp recordings were performed on mEC or BC neurons with glass pipettes pulled from standard borosilicate glass, with a resistance of 3–5 MΩ. The pipette solution used for current clamp experiments consisted of 110 mM potassium gluconate, 40 mM HEPES, 2 mM ATP-Mg, 0.3 mM GTP, and 4 mM NaCl (pH 7.2–7.3, 280–290 mOsm L^−1^). Voltage clamp experiments used pipette solution composed of 140 mM CsCl, 0.2 mM EGTA, 10 mM HEPES, 5 mM QX-314, 0.3 mM GTP, 2 mM Mg-ATP (pH 7.2–7.3, 280–290 mOsm L^−1^). Liquid junction potential was not corrected for. For current clamp, no holding current was used, and cells with a membrane potential positive to −55 mV during Down states or a change in input resistance greater than 20% in the first 10-min control recording were excluded. For voltage clamp recordings, a stable series resistance below 10 MΩ during the first 10 min was required before applying series resistance compensation. When stimulation was used to evoke Up states or synaptic currents, a stimulation intensity of 50–250 μA and pulse duration of 10–40 μs were used unless otherwise stated. The stainless steel stimulating electrode was placed in the same layer as the recording electrode in voltage clamp experiments; in Up state experiments it was placed in layer III of the mEC and layer V of the BC, as these layers are understood to generate spontaneous Up state activity (Cunningham et al., 2006, Tahvildari et al., 2012, Beltramo et al., 2013, Stroh et al., 2013). In all cases, only a single pulse was used in order to evoke synaptic currents or Up state events.

### 2.3 Acquisition and analysis

All data were collected using an Axon Multiclamp 700B amplifier (Molecular Devices, Sunnyvale, CA, USA), low-pass filtered at 2 kHz and digitized at 5 kHz using an Instrutech ITC-18 A/D board and custom-made acquisition procedures in Igor Pro (Wavemetrics). Custom procedures within Igor Pro were also used to analyse the data. Up states were detected using a previously published algorithm that uses changes in the membrane potential and voltage noise to detect transitions between Up and Down states (Craig et al., 2013). The detected state transitions were all confirmed by visual inspection.

Synaptic charge transfer was estimated by integrating the original current trace from the stimulus artefact to the return to baseline on traces low-pass filtered at 50 Hz. The time point of each decile of charge transfer was plotted to compare the temporal profile of synaptic currents (see Fig. 6B). To the estimate the charge transfer of the slow component of synaptic current as a fraction of the total synaptic charge transfer, for each experiment, a double exponential function was fitted to the decaying phase of the EPSC from the peak to 200 ms after the stimulation artefact. If neither component had a decay time constant exceeding 20 ms, a single exponential function was fitted and only a fast component was considered present.

The charge transfers of the fast and slow component were estimated as the sum of the integral under the respective fitted exponential curves and the integral of the rising phase estimated as a linear function from the start of the EPSC to the start of the decaying exponential function, using the built-in Integrate function in Igor Pro. The total charge transfer was estimated as the sum of the charge transfer values for each component.

The IC50 for GYKI-53655 was estimated using the Hill equation (see Fig. 5B).

Confidence intervals for current traces were computed in Igor Pro using the built-in Average Waves function.

### 2.4 Chemicals

1-(4-Aminophenyl)-3-methylcarbamyl-4-methyl-3,4-dihydro-7,8-methylenedioxy-5*H*-2,3-benzo–diazepine hydrochloride (GYKI-53655), (S)-1-(2-amino-2-carboxyethyl)-3-(2-carboxybenzyl)pyrimidine-2,4-dione (UBP-302), 6-cyano-7-nitroquinoxaline-2,3-dione (CNQX) and 6-imino-3-(4-methoxyphenyl)-1(6H)-pyridazinebutanoic acid hydrobromide (gabazine) were supplied by Tocris. d-(-)-2-Amino-5-phosphonopentanoic acid (d-AP5) was purchased from Abcam. Drugs were applied by dissolving them into circulating aCSF at the desired concentration. UBP-302 was dissolved in dimethyl sulfoxide (DMSO) supplied by Tocris (DMSO at 0.02% in circulated solution). A wash-in duration of at least 5 min was allowed for all drugs before the effects were evaluated.

### 2.5 Statistics

All statistics were performed on raw data using Prism 5 (GraphPad Software Inc.). Paired and unpaired two-tailed Student's *t*-tests as appropriate were used in order to compare data in different conditions, as well as one-way and repeated-measures (RM) ANOVA with post-hoc Bonferroni multiple-comparison for comparisons of more than two means. Results were considered significant when *p* < 0.05. All values are given as the mean ± S.E.M.

### 2.6 Neuronal network model

Layer III of the mEC was modelled with 100 excitatory cells (E cells) and 10 fast-spiking inhibitory cells (I cells) with intrinsic properties as shown in Table 1, and with connection probabilities of 0.1 between E cells, and 0.5 from E to I cells as well as from I to E cells, with synaptic parameters in accordance with experimental observations of Dhillon and Jones (2000) and Cunningham et al. (2006) (Table 2). Single-neuron behaviour was simulated by an exponential integrate-and-fire model (Gerster et al., 2014) with adaptation provided by an exponentially decaying potassium conductance,

$$C\frac{\mathrm{d}V}{\mathrm{d}t}=-g_{L}\left(V-V_{L}\right)+g_{L}\Delta \text{exp}\left(\frac{V-V_{\mathrm{Thres}}}{\Delta }\right)-g_{\mathrm{Ad}}\left(V-V_{\mathrm{Ad}}\right)+I_{\mathrm{sym}}$$

$$\frac{\mathrm{d}g_{\mathrm{Ad}}}{\mathrm{d}t}=-\frac{g_{\mathrm{Ad}}}{\tau_{\mathrm{Ad}}}$$

$$\text{If}\;V>\theta_{\mathrm{Thres}}\Rightarrow V\to V_{\mathrm{reset}};g_{\mathrm{Ad}}\to g_{\mathrm{Ad}}+\Delta g_{\mathrm{Ad}}$$

$$I_{\mathrm{sym}}^{k}={\text{GABA}}_{B}\text{factor}\sum_{i}^{\mathrm{exc}}\text{Conn}\left[i,k\right]\left(I_{\text{AMPA}}^{i}+I_{\text{KA}}^{i}\right)-\sum_{j}^{\mathrm{inh}}\text{Conn}\left[j,k\right]I_{{\text{GABA}}_{A}}^{j}$$

Ix=−gx(V−Vx);gx=gmax,xτxe−1τx

where *C* is capacitance, *V* is membrane potential, *g*_*L*_ and *V*_*L*_ are leak conductance and reversal potential, *g*_*Ad*_, *V*_*Ad*_, and *τ*_*Ad*_ are the conductance, reversal potential, and decay constant of the adaptation current. An action potential occurs when *V* crosses the value *θ*_*Thres*_, following which *V* is reset to *V*_*reset*_ and *g*_*Ad*_ is increased by Δ*g*_*Ad*_.

$I_{\mathrm{sym}}^{k}$ is the total synaptic input to neuron *k*, calculated as a sum of individual currents from contributing neurons. Conn[*i*,*k*] is 1 if neuron *i* is connected to neuron k and 0 if not. Three synaptic currents were considered, GABA_*A*_, AMPA, and KA, simulated with exponentially decaying conductances. *I*_*x*_ is a current of type *x* generated by one synapse with conductance *g*_*x*_, reversal potential *V*_*x*_, and decay time constant *τ*_*x*_. Maximal conductances *g*_max,*x*_ were adjusted to reproduce the observed network dynamics (Table 2).

Presynaptic GABA_B_ receptors (limiting the release of glutamate as a response to build-up of spillover GABA; Craig et al., 2013) were modelled by multiplying the excitatory currents by a factor that could take values between 0 and 1, shaped as a sigmoidal function:

GABABfactor=11+eA(−1+GABAcounter/B)

where *A* = 8 and *B* = 1500 are constants, regulating the sharpness of an Up-to Down-state transition and the duration of the Up state respectively. The GABA counter was reduced by 1 per ms time elapsed during Down states. Input from other brain areas was modelled as stochastic activation of pyramidal cells every 2 ms with a probability of 0.5% per neuron.

## 3 Results

### 3.1 Spontaneous mEC Up states outlast BC Up states

**Fig. 1:**
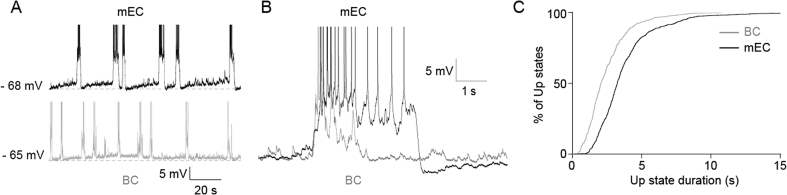
mEC Up states *in vitro* outlast those in neocortex. (A) Current clamp recordings from mEC layer III and BC layer V principal neurons in separate slices. (B) Enlargement of superimposed individual Up states from mEC and BC. (C) Cumulative distribution of Up state duration in control conditions in mEC and BC (mEC, 614 Up states from 31 slices; BC, 330 Up states from 30 slices).

mEC Up states outlast neocortical Up states *in vivo* (Hahn et al., 2012). In order to investigate whether this difference might be explained by intrinsic circuit differences between these cortical areas, we compared spontaneous Up state properties recorded from individual principal neurons in slices from the mEC and the BC as a representative of neocortex. Up state properties are listed in Table 3. Spontaneous Up state duration across all experiments in layer III of the mEC was 4.24 ± 0.28 s (n = 31, Fig. 1A–C; Table 3), while in layer III and V of the BC the Up state duration was 2.97 ± 0.25 s (n = 15) and 2.21 ± 0.34 s (n = 15, Fig. 1A and B; Table 3), respectively. There was no significant difference between the mean Up state durations in the BC layers (p = 0.09). The mean Up state duration in layer III of the mEC was significantly longer than that of the pooled Up states across layers III and V of the BC (2.60 ± 0.22 s, n = 30, p = 0.00003, Fig. 1C), as well as the mean from each layer taken individually (p = 0.006 compared with BC layer III, p = 0.00009 compared with BC layer V). The relative longevity of the mEC Up state (Fig. 1B and C) is consistent with the *in vivo* findings of Hahn et al. (2012). We next asked what mechanisms might underlie this difference between cortical areas.

### 3.2 Effects of NMDA receptor blockade on spontaneous Up states in the mEC and BC

**Fig. 2:**
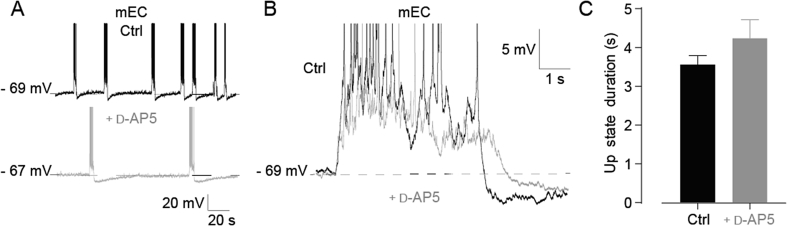
NMDA receptor blockade has no effect on Up state duration in mEC layer III. (A) Up state recordings in control conditions and after application of 50 μM d-AP5 in mEC. (B) Superimposed Up states from mEC in control condition (black), and after bath application of d-AP5 (grey). (C) Histogram showing mean Up state duration in mEC layer III in control conditions and after bath application of d-AP5. Note that d-AP5 has no effect on Up state duration. Error bars indicate s.e.m., n = 5.

Up states are maintained by synaptic glutamate receptors (Sanchez-Vives and McCormick, 2000, Shu et al., 2003). We asked whether different involvement of glutamate receptors might be responsible for the distinct properties of BC and mEC Up states, focusing first on NMDA receptors, which have been reported to be required for Up states in the neocortex (Sanchez-Vives and McCormick, 2000, Shu et al., 2003, Castro-Alamancos and Favero, 2015). Consistent with this, all Up state activity was abolished by the NMDA receptor antagonist d-AP5 (50 μM) in 5 BC slices recorded in layer III, and 5 BC slices recorded in layer V. In contrast, in layer III of the mEC, Up states were preserved, and their duration showed no significant change following d-AP5 application (control, 3.56 ± 0.23 s; d-AP5, 4.24 ± 0.48 s; p = 0.118, n = 5, Fig. 2A–C). Up state incidence, however, did fall significantly (control 0.069 ± 0.0098 Hz; d-AP5, 0.010 ± 0.0039 Hz, p = 0.0038, n = 5, Table 4). This highlights the importance of NMDA receptors for Up states in the BC, while suggesting that the mEC Up state may rely on different synaptic mechanisms.

### 3.3 Effects of kainate receptor blockade on spontaneous Up states in mEC and BC

**Fig. 3:**
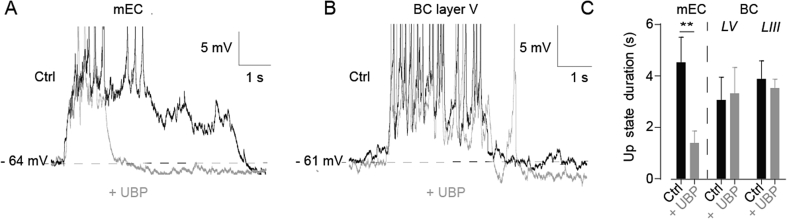
Kainate receptor blockade shortens mEC Up states but not BC Up states. (A) Superimposed mEC layer III Up states in control conditions (black) and after bath application of 20 μM UBP-302 (grey). (B) Superimposed BC layer V Up states in control conditions (black) and after bath application of UBP-302 (grey). (C) Histograms showing mean Up state duration in control conditions and after bath application of UBP-302 in mEC layer III, and BC layers V and III. Note that UBP-302 significantly reduces Up state duration in mEC layer III, but has no effect in either layer of the BC. Error bars indicate s.e.m, n = 5; **, p < 0.01, paired *t*-test.

We next investigated the involvement of kainate receptors, since mEC Up states are generated in layer III of the mEC (Cunningham et al., 2006), and kainate receptors have been reported to be expressed specifically in principal neurons from the superficial layers of the mEC (West et al., 2007). We used UBP-302 (20 μM) to block kainate receptor currents. This abolished Up states in two of seven mEC slices. In the remaining five slices Up state duration was significantly shortened from 5.37 ± 0.93 s (n = 5) in control conditions to 1.99 ± 0.20 s following application of UBP-302 (n = 5; p = 0.014; Fig. 3A, C). By contrast, in layers III and V of the BC no significant change in Up state duration was observed in response to application of UBP-302. In BC layer V, Up state duration was 3.06 ± 0.89 s in control conditions and 3.32 ± 1.01 s following UBP-302 application (p = 0.61; n = 5; Fig. 3B and C) and in BC layer III, 3.88 ± 0.70 s in control conditions and 3.53 ± 0.34 s following UBP-302 application (p = 0.53; n = 5; Fig. 3C). These results indicate that kainate receptors are important for the maintenance of Up states in the mEC, but not in the BC.

### 3.4 Effects of AMPA, NMDA and kainate receptor blockade on the maintenance of evoked Up states in the mEC and BC

**Fig. 4:**
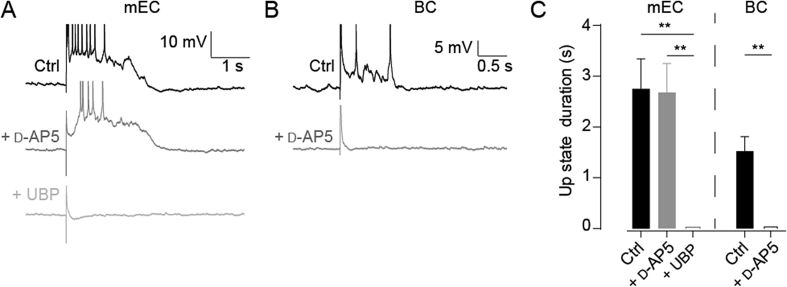
Sequential NMDA and kainate receptor blockade abolishes evoked Up states in mEC layer III. (A) Sample traces from a cell of mEC layer III in control conditions (black), after bath application of 50 μM d-AP5 (dark grey) and after addition of 20 μM UBP-302 (light grey). (B) Sample traces from BC layer V in control conditions (black) and after bath application of d-AP5 (dark grey). (C) Histograms showing mean Up state duration in control conditions, and after d-AP5 and UBP-302 application in mEC layer III, and after d-AP5 application in BC layer V. Notice that, as with spontaneous Up states, d-AP5 alone has no effect on Up state duration in mEC layer III while subsequent UBP-302 application abolished evoked Up states entirely. In contrast, d-AP5 application alone completely abolished evoked Up states in BC layer V. Error bars indicate s.e.m., n = 5; **, p < 0.01. RM-ANOVA and Bonferroni post hoc test.

In order to investigate the effect of glutamate receptor antagonists on maintenance of Up states isolated from the effect on spontaneous incidence, Up states were evoked by electrical stimulation. No significant difference was found in evoked Up state duration following d-AP5 application in the mEC (control, 2.75 ± 0.59 s versus d-AP5, 2.68 ± 0.57 s; p = 0.801, n = 5; Fig. 4A and C). However, the rate of successful stimulation did fall significantly (successful stimulations/attempted stimulations; control, 0.867 ± 0.084; d-AP5, 0.353 ± 0.155, p = 0.032, n = 5; 32 trials per cell on average, stimulating electrode position kept constant between all trials). In all of these recorded cells, subsequent application of UBP-302 prevented any evoked Up state activity (Fig. 4A and C), even after incremental increases in stimulus intensity up to 1.5 mA (not shown). These results suggest that AMPA receptor currents alone are not capable of maintaining the Up state in the mEC, without the assistance of either kainate or NMDA receptor activation. In the BC, NMDA receptor blockade fully prevented electrical induction of Up states (control duration: 1.53 ± 0.29 s, n = 5; Fig. 4B and C), even when stimulus intensity was increased incrementally up to 1.5 mA. This finding confirms the necessity of NMDA receptor-mediated currents in the maintenance of Up states in the BC.

**Fig. 5:**
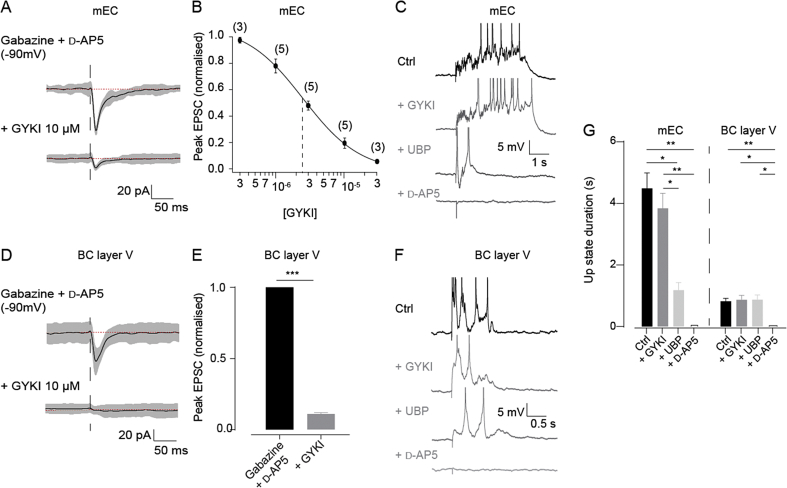
The effect of sequential blockade of AMPA, kainate and NMDA receptors in mEC layer III and BC layer V. (A) Average evoked synaptic current (black line) in mEC layer III in the presence of 3 μM gabazine and 50 μM d-AP5 before (top) and after (bottom) application of 10 μM GYKI-53655 (grey shaded area, 95% confidence interval, red dotted lines correspond to the mean current before the stimulation). (B). Dose-response relationship of GYKI-53655 on evoked AMPA current. The approximate IC50 was 2.5 μM (solid line). At 10 μM, the concentration used in the main experiments, GYKI-53655 blocked 80.5% ± 3.9% of the peak current (n = 5). These results are similar to previous reports regarding the potency of GYKI-53655 (Paternain et al., 1995). (C) Evoked Up states in mEC, in control conditions (black) and after the application of 10 μM GYKI-53655, and subsequent application of 20 μM UBP-302, and 50 μM d-AP5 (grey). (D) Average evoked synaptic current (black line) in BC layer V (grey thresholds: 95% confidence interval, red dotted lines correspond to the mean current before the stimulation). (E) Histogram of the normalised peak evoked synaptic current before and after application of GYKI-53655. Error bars indicate s.e.m., n = 5; ***, p < 0.001, paired *t*-test. (F) Sample traces from BC in control conditions (black), and after sequential application of GYKI-53655, UBP-302, and then d-AP5 (grey). (G) Histograms of Up state duration in mEC layer III and BC layer V in control conditions and after the sequential blockade of AMPA, kainate and NMDA receptors. Notice that no significant effect was seen on Up state duration in BC layer V until application of d-AP5, whereupon evoked Up states were abolished. Conversely, in mEC layer III, while GYKI-53655 had no significant effect on duration, UBP-302 significantly reduced it. Subsequent application of d-AP5 abolished Up states in mEC. Error bars indicate s.e.m., n = 5; *, p < 0.05, **, p < 0.01, ***, p < 0.001. RM-ANOVA and Bonferroni post hoc test. (For interpretation of the references to colour in this figure legend, the reader is referred to the web version of this article.)

In order to investigate a possible role for AMPA receptors in Up state maintenance, we used the moderately selective AMPA receptor antagonist GYKI-53655. GYKI-53655 (10 μM) blocked more than 80% of the synaptically evoked peak inward current in mEC layer III neurons (IC50, 2.5 μM; Fig. 5A and B) and 89% in BC layer V (Fig. 5D and E). Bath application of 10 μM GYKI-53655 did not significantly affect the duration of evoked Up states in the mEC (control: 4.48 ± 0.61 s vs GYKI-53655: 3.83 ± 0.43 s, n = 5, p > 0.05; Fig. 5C and G), but subsequent superfusion of 20 μM UBP-302 drastically reduced their duration (1.18 ± 0.22 s, n = 5, p < 0.01 when compared to GYKI-53655; Fig. 5C and G). Additional NMDA receptor blockade by 50 μM d-AP5 entirely abolished evoked Up states (Fig. 5C and G). Similarly, the duration of evoked Up states in the BC was not altered by 10 μM GYKI-53655 (control, 0.81 ± 0.1 s vs GYKI, 0.86 ± 0.14 s, n = 5, p = 0.99 Fig. 5F and G). Unlike the mEC, Up states in the BC were not altered by addition of 20 μM UBP-302 (0.86 ± 0.15 s, n = 5, p = 0.99; Fig. 5F and G), but were completely blocked by 50 μM d-AP5 (0 s, n = 5, p = 0.03; Fig. 5F and G), Supporting the conclusion that AMPA receptors are not required for the maintenance of Up states, Up states could still be evoked in the BC following complete blockade of non-NMDA ionotropic glutamate receptors with 10 μM CNQX, consistent with previous reports (Favero and Castro-Alamancos, 2013). Together, these findings suggest that AMPA receptors are not required for the maintenance of Up states in either mEC or BC and that the persistence of the mEC Up state is mediated by kainate receptors, while NMDA receptors are required for Up state maintenance in the BC and for the remaining evoked Up states following kainate receptor block in the mEC.

### 3.5 Presence of a non-NMDA glutamate receptor-mediated slow current in mEC but not in BC

**Fig. 6:**
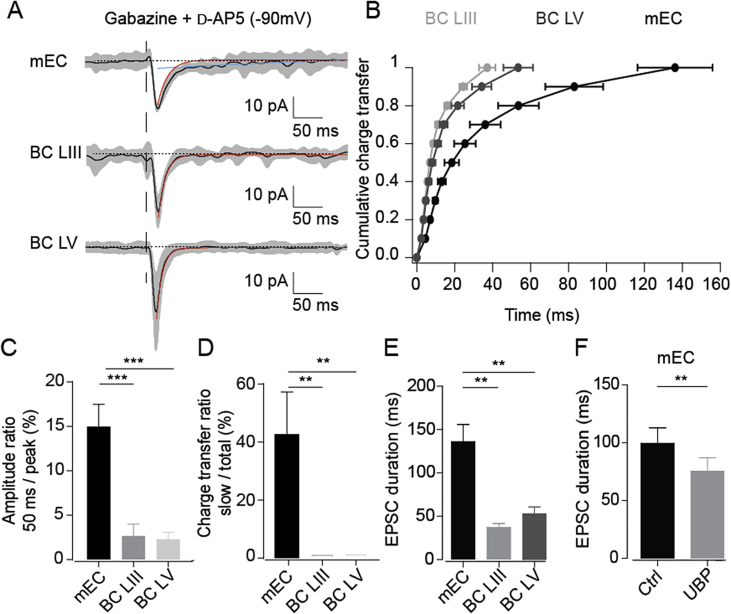
A prominent non-NMDA slow current in mEC layer III compared to BC layer III and V. (A) Average evoked synaptic currents (black trace) in mEC, BC layer III and layer V (grey contours: 95% confidence interval, black dotted lines correspond to the mean current before the stimulation). The red exponential fitted curve shows a fast component in BC and mEC while only mEC exhibits a slow non-NMDA component (blue exponential fitted curve). (B) Normalised cumulative charge transfer of evoked synaptic currents as a function of time. n = 5. (C) Amplitude of evoked synaptic current 50 ms after stimulation relative to peak amplitude in mEC layer III, and BC layers V and III. Note the much larger remaining current in mEC layer III at the 50 ms time point, indicating higher kainate/AMPA ratio. n = 5; ***, p < 0.001, unpaired *t*-test. (D) Slow charge transfer in percent of total charge transfer. Note that the slow charge transfer accounts for more than 40% of the total in mEC. n = 5; **, p < 0.01, unpaired *t*-test. (E) Duration of EPSC in mEC, BC layer III and layer V. n = 5; **, p < 0.01, unpaired *t*-test. (F) EPSC duration in mEC layer III in control condition and after application of 20 μM UBP-302. n = 5; **, p < 0.01, paired *t*-test. Note that UBP-302 significantly reduced the slow component of the EPSC in the mEC. All error bars indicate s.e.m. (For interpretation of the references to colour in this figure legend, the reader is referred to the web version of this article.)

To investigate whether the above effects could be due to the presence of postsynaptic kainate receptors, we next addressed the question whether mEC neurons expressed a different ratio of kainate/AMPA receptor-mediated synaptic currents compared to BC neurons that would confer to the mEC this persistent Up state activity. For that, we performed voltage clamp recordings of neurons from mEC layer III, as well as BC layers III and V in the presence of GABA_A_ and NMDA receptor blockers (3 μM gabazine and 50 μM d-AP5). These experiments were performed in aCSF of a different composition (see Materials and Methods, Section 2.1) in order to reduce the generation of network events upon electrical stimulation. Non-NMDA excitatory currents were evoked by electrical extracellular stimulation in the same layer at a holding potential of −90 mV. Evoked excitatory currents in the mEC displayed significantly slower kinetics than the currents evoked in layers III or V of the BC (Fig. 6A). The time of the last decile of the mEC currents was 136 ± 20 ms while those in layers III and V of the BC were 37 ± 10 and 53 ± 10 ms, respectively (n = 5, **p < 0.01, one-way ANOVA, Fig. 6B). In order to obtain an estimate of the ratio of kainate/AMPA currents, the current amplitude was measured 50 ms after the stimulation pulse, a time point at which AMPA receptor-mediated currents fall to below 5% (Kohl et al., 2011). Normalised to the EPSC peak amplitude, mEC layer III cells had a current amplitude at 50 ms of 15.0 ± 2.5% (n = 5), while BC layer V and BC layer III had current amplitudes at 50 ms of 1.9 ± 1.6% (n = 5) and 0.2 ± 1.4% (n = 5), respectively (Fig. 6C). The mECIII current ratio was significantly different from BC layer V and BC layer III (p = 0.0008 and p = 0.0006 respectively), while there was no difference between BC layer V and BC layer III (p = 0.99). Furthermore, charge analysis revealed that non-NMDA glutamate receptor-mediated slow current components were present in 4 out of 5 cells in the mEC, while no cells in BC layer V or BC layer III had a detectable non-NMDA glutamate receptor-mediated slow current component. In mEC layer III, the fast time constant of decay (tau) was 11 ± 1 ms, while the slow tau was 65 ± 13 ms. The proportion of charge transferred by this slow current component was 42 ± 14% (n = 5; Fig. 6D). This result suggests a greater kainate/AMPA ratio in the mEC than in BC, and shows that a large proportion of the total charge transferred by synaptic events in the mEC is mediated by a non-NMDA glutamate receptor-mediated slow current component.

To confirm that the slow excitatory current observed in the mEC is indeed mediated by kainate receptors, we applied UBP-302 (20 μM), which shortens the duration of mEC Up states (Fig. 3A and C), in the presence of d-AP5 and gabazine, at a holding potential of −70 mV. The EPSC duration was significantly longer in mEC than in BC layer III and BC layer V (Fig. 6E), and UBP-302 significantly shortened the EPSC duration in the mEC from 99 ± 13 ms to 75 ± 12 ms (n = 5; p = 0.006; Fig. 6F), and reduced the charge ratio 42 ± 14% to 27 ± 4% (n = 4). This UBP-302 sensitivity confirms that a significant proportion of the slow non-NMDA glutamate receptor-mediated current observed in the mEC is mediated by kainate receptors.

### 3.6 Functional implications of kainate receptor-maintained Up states in mEC

**Fig. 7:**
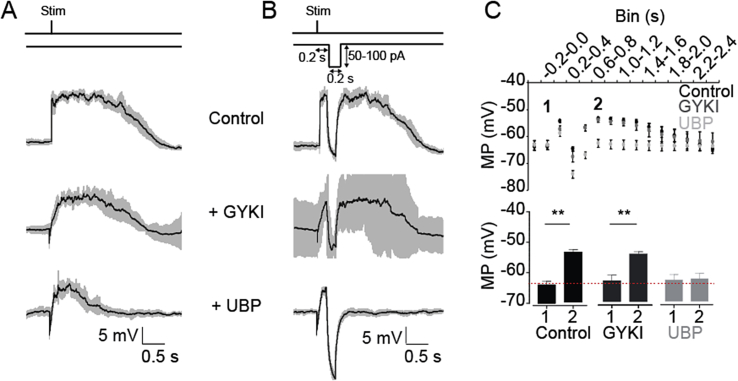
Kainate receptor-mediated currents are necessary to recover from hyperpolarisation during an Up state in mEC. (A, B) Stimulating and hyperpolarising protocol used in alternation. Average evoked Up state traces (black lines) in control (A) or with hyperpolarisation (B) and 95% confidence interval (grey shaded area). (C) Average membrane potential binned in 200 ms time periods during evoked Up state with hyperpolarisation in drug-free condition (black), in the presence of 10 μM GYKI-53655 (dark grey) and with the further addition of 20 μM UBP-302 (light grey). The red dotted line represents the average membrane potential before stimulation in control condition. Error bars indicate s.e.m., n = 5; **, p < 0.01, one-way ANOVA or paired *t*-test. (For interpretation of the references to colour in this figure legend, the reader is referred to the web version of this article.)

To explore possible functional implications of the involvement of kainate receptors in the maintenance of persistent Up states in the mEC, we compared the effect of a hyperpolarising current step in principal neurons in layer III of the mEC during evoked Up states. Utilising a hyperpolarising current step in order to interrupt Up state activity *in vitro* has been performed previously (Oikonomou et al., 2012). Because of their different current-voltage relationships, we predicted that Up states in individual cells driven predominantly by NMDA receptors would be more easily terminated by hyperpolarisation than those driven by kainate receptors. A hyperpolarising current step tuned to bring the membrane potential just negative to the Down state potential in the control condition was applied 200 ms after evoking an Up state (see protocol Fig. 7B). Up states with and without hyperpolarisation were alternated, and then subsequently grouped and averaged, such that an average Up state with and without hyperpolarisation was plotted with the corresponding 95% confidence interval (Fig. 7A and B).

Following the release from the hyperpolarising current, the membrane potential of mEC layer III cells rebounded to a level comparable to the Up state membrane potential (Down state membrane potential: −63.5 ± 0.7 mV, membrane potential 200–400 ms after end of hyperpolarising pulse: −53.2 ± 1.0 mV, n = 5, p < 0.05, Fig. 7C). Similarly, after application of 10 μM GYKI-53655, the membrane potential recovered to similar levels (Down state membrane potential: −62.7 ± 0.8 mV, membrane potential 200–400 ms after end of hyperpolarising pulse: −53.2 ± 1.3 mV, n = 5, p < 0.05, Fig. 7C). However, following application of 20 μM UBP-302, using the same current amplitude, the membrane potential relaxed back to the Down state membrane potential (Down state membrane potential: −62.5 ± 0.8 mV, membrane potential 200–400 ms after end of hyperpolarising pulse: −62 ± 1 mV, n = 5, p > 0.05, Fig. 7C). These data may help explain how kainate receptors can maintain the Up state in mEC neurons beyond the neocortical Up state. Unfortunately, equivalent experiments were not possible in the BC, because the Up state duration in control conditions was much shorter (Fig. 5F and G).

### 3.7 Mechanism of prolonged Up states in the mEC

**Fig. 8:**
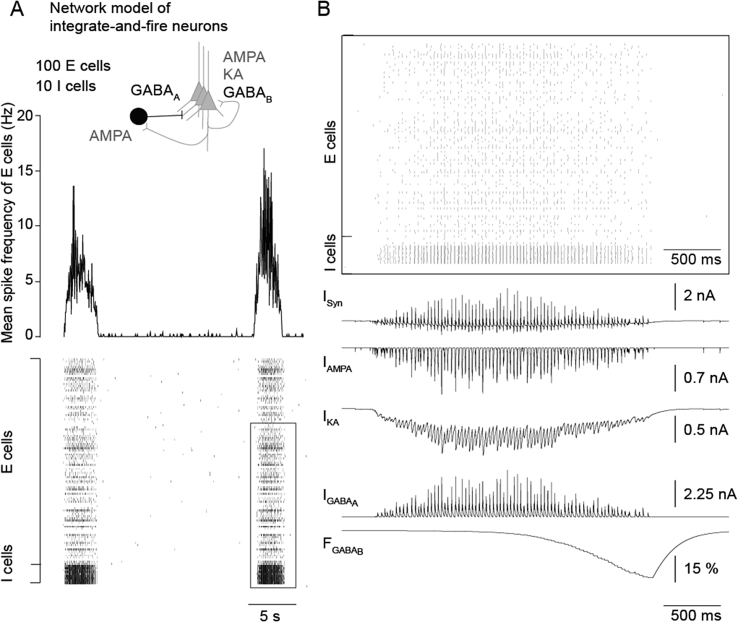
Kainate receptor-mediated currents are sufficient to maintain persistent mEC Up states. (A) Inset: Schematic of the neuronal network model and the receptors involved. Average spiking frequency from the excitatory neurons (E cells) as well as raster plot of excitatory and inhibitory neurons (I cells). Note that the network generates Up state activity interrupted by Down state periods when only few neurons are spiking. (B) Detail corresponding to the black frame in the raster plot and the synaptic resultant synaptic currents (I_syn_), AMPA (I_AMPA_), kainate (I_KA_), and GABA_A_ currents (I_GABAA_) as well as the GABA_B_ factor (F_GABAB_).

In order to investigate the sufficiency of kainate receptor-mediated currents in maintaining Up states in the mEC, we built a computational model of a recurrent network of exponential integrate-and-fire neurons. Excitation was mediated by AMPA and kainate receptors, while inhibition was modelled by GABA_A_ and GABA_B_ receptors (Fig. 8A). In the model, feedback between sparsely firing pyramidal neurons and the GABAergic interneuron pool generated gamma oscillations via GABA_A_ receptors (Fig. 8B), while the Down state transition was mediated by presynaptic GABA_B_ receptors at excitatory synapses activated by the accumulated firing of inhibitory neurons (Fig. 8B; Craig et al., 2013).

In accordance with our voltage clamp recordings, the decay time constant of kainate receptor-mediated currents was set to 100 ms. This experimental parameter was included in our model to see if we were able to generate Up states. Indeed, due to this long decay time constant, the kainate receptor-mediated component maintains this persistent network activity, as the currents remain active across the gamma oscillation cycles (See Fig. 8B). In our simulation, the Up state duration, controlled by GABA_B_ receptors, exceeded 3 s (Fig. 8A and B). In the absence of NMDA receptor-mediated currents and specific intrinsic properties, we hence demonstrate that the kainate receptor-mediated current is sufficient to maintain Up state activity.

## 4 Discussion

We have examined the different mechanisms involved in the maintenance of Up states in the mEC and BC. Kainate receptor-mediated currents are the main contributor to the persistent phase of Up states in the mEC, while NMDA receptors are crucial for the maintenance of Up states in the BC. The presence of postsynaptic kainate receptor-mediated currents in the mEC was confirmed and, importantly, similar currents were not observed in principal neurons of either layer III or V of the BC. The post-synaptic kainate receptor-mediated currents have the functional implication that they assist the recovery to the Up state membrane potential following hyperpolarising inputs. Finally, we show in a network model of recurrently connected neurons that the kainate receptor-mediated current is sufficient to mediate a persistently active network state.

### 4.1 The mechanisms underlying maintenance of the Up state are different in the entorhinal and barrel cortices

The Up state duration in the mEC was significantly longer than in the BC, when compared to layer III or layer V individually, or all BC Up states pooled. This finding is in agreement with the *in vivo* study of Hahn et al. (2012) and implies that the mechanism underlying the longer Up state duration resides intrinsically within the mEC itself. Our evidence suggests that this mechanism involves kainate receptors. Indeed, in the presence of UBP-302, the Up states of the mEC were of similar duration to those of the BC.

Similar to a previous study in the rat mEC (Cunningham et al., 2006), NMDA receptor antagonists did not block Up state generation. However, in contrast to this study, in which UBP-302 abolished Up states, in our experiments, although kainate receptor blockade significantly reduced Up state incidence (Table 4), it did not abolish them. This could be due to species differences, or slightly different experimental conditions, such as aCSF composition. Moreover, our experiments included electrically evoked Up states, enabling a distinction between a network that could no longer maintain Up state activity and a network that simply lacked sufficient excitation to initiate Up states. mEC Up states could still be evoked in the presence of UBP-302, albeit they were of significantly shorter duration, suggesting that, although they are not necessary, NMDA receptors are sufficient to maintain Up states also in the mEC, but kainate receptors are required for their longer duration.

In contrast to the mEC, NMDA receptor blockade completely abolished both spontaneous and evoked Up state activity of the BC. This result is consistent with many previous reports in neocortical slices (Sanchez-Vives and McCormick, 2000, Shu et al., 2003, Castro-Alamancos and Favero, 2015), This may appear surprising given that animals anaesthetised with ketamine, known to be an NMDA receptor antagonist, still show Up state activity (Ruiz-Mejias et al., 2011, Sheroziya and Timofeev, 2014). Nevertheless, in cats anaesthetised with urethane, subsequent application of ketamine reduced slow oscillation Up state duration but increased their incidence, showing that *in vivo* Up states are sensitive to NMDA receptor blockade (Steriade et al., 1993b). The apparent discrepancy of abolition versus modification could be due to a number of factors, including, firstly, that the network connectivity in slices is necessarily restricted, as connections to other cortices and subcortical structures are removed, and secondly, the ketamine dosage necessary to maintain anaesthesia is unlikely to block all NMDA receptor currents, unlike d-AP5 at 50 μM. Thus, the presence of Up states during ketamine anaesthesia *in vivo* does not undermine the fundamental difference that we report; the mEC local network Up state is not affected by NMDA receptor blockade, but the BC Up state is.

### 4.2 The “persistent” Up state of the mEC is maintained by kainate receptor currents

Results of experiments in which a hyperpolarising step of 200 ms was delivered within the duration of the Up state further elucidate the mechanisms involved in the mEC Up state persistence. As predicted if postsynaptic kainate receptors were involved, the mEC principal cells were capable of rebounding back to the Up membrane potential upon release of the hyperpolarising step, as both AMPA and kainate receptors show linear I/V relationships at physiological negative potentials (Cui and Mayer, 1999). As such, the hyperpolarisation would increase the magnitude of these currents and aid recovery to the Up state. The cell still recovered to the Up membrane potential consistently during AMPA receptor blockade with GYKI-53655. In contrast, following block of kainate receptors with UBP-302, the cell did not recover to the Up state following hyperpolarisation, suggesting that kainate receptors are indeed responsible. The non-linear I/V properties of NMDA receptors prevent their involvement in membrane potential recovery following hyperpolarisation.

### 4.3 Functional significance of a kainate receptor-maintained “persistent” Up state

The presence of postsynaptic kainate receptors in addition to NMDA receptor-mediated currents in mEC layer III neurons is of functional significance when considered alongside models of Up state termination dependent on GABA_B_ receptors (Mann et al., 2009, Craig et al., 2013). This is especially relevant as postsynaptic GABA_B1b_ receptor activation results in hyperpolarisation of the target cell (Bettler et al., 2004). NMDA receptor-mediated currents reduce in amplitude with hyperpolarisation negative to −40 mV, while kainate receptor-mediated currents increase linearly with hyperpolarisation in the physiological range (Cui and Mayer, 1999). Therefore, hyperpolarisation of cells expressing predominantly NMDA receptors would reduce the slow component of the postsynaptic inward current, while hyperpolarisation of cells expressing predominantly kainate receptors would increase it. The former mechanism would yield bistability of individual cells during Up states, whereas the second mechanism could help explain why the mEC Up state outlasts neocortical Up states *in vivo* (Hahn et al., 2012). This also has implications for the mechanism of Up state termination in the mEC as previously suggested (Craig et al., 2013). Conceivably, because of the resilience to hyperpolarisation that the kainate receptor-mediated component provides, the most effective ways to terminate the mEC Up state are by reduction in transmitter release via presynaptic GABA_B1a_ receptors, or by the long-duration hyperpolarisation provided by postsynaptic GABA_B1b_ receptors. The GABA_B_ receptor model of Up state termination has yet to be demonstrated *in vivo*. The kainate receptor-mediated components may, however, make individual cells of the mEC less likely to drop out of the Up state when considering other proposed Up state termination mechanisms, such as the build up of outward K^+^ conductances (Sanchez-Vives and McCormick, 2000, Cunningham et al., 2006). This could lead to persistence of the Up state in the mEC beyond NMDA receptor-driven neocortical Up states.

### 4.4 Computational insights

Our computational model was kept as simple as possible, whilst maintaining physiological relevance; this included the use of exponential integrate-and-fire neurons with minimal intrinsic properties and restriction of synaptic currents to only AMPA, kainate and GABA_A_ receptors. The objective of the model was to demonstrate the sufficiency of kainate receptors in the maintenance of Up state activity; this objective was satisfied, as no external tonic excitation or other slow currents were present, and yet the Up state was maintained.

In the computational model, the kainate receptors generate a tonic excitation that lasts throughout the Up state. The slow decay of these currents enables persistence across gamma cycles. This is consistent with the experimental finding that removal of all slow synaptic components in the mEC abolishes Up states entirely; without this slow component, the network could not sustain a persistently active state. As the blockade of only one slow component in the mEC preserves Up state activity, albeit significantly reduced in duration in the case of kainate receptor blockade, it therefore is likely that the mEC network utilises slow components provided by both NMDA and kainate receptors; while the BC network requires NMDA receptor-mediated currents to maintain the Up state. This is further supported by the similarity of Up state durations observed between control Up states in the BC and Up states following kainate receptor blockade in the mEC; in these conditions, both networks rely on NMDA receptor-mediated transmission to provide Up state maintenance.

### 4.5 Comparison to *in vivo* Up states

Slice models of Up states have been used extensively, and successfully, to explore fundamental mechanisms of Up state generation and termination (Sanchez-Vives and McCormick, 2000, Cossart et al., 2003, Cunningham et al., 2006, Mann et al., 2009, Craig et al., 2013, Castro-Alamancos and Favero, 2015). However, it is necessary to consider that other features of Up states, such as interactions with other brain regions, can only be observed *in vivo*. Such studies regularly report an Up state incidence that is higher than we report here (Hahn et al., 2012, Chauvette et al., 2010). This is unsurprising, as the network is both denser and more expansive *in vivo*, covering more than a single cortical region. However, concerning Up state duration, that found *in vivo* is considerably more similar to that recorded *in vitro*. Coupled with the fact that Up states in the mEC outlast neocortical Up states both *in vivo* and *in vitro*, the mechanistic findings reported here may well be relevant for Up states *in vivo*.

### 4.6 The effects of AMPA receptor blockade

The finding that AMPA receptor blockade does not prevent the maintenance of Up states may seem surprising, given the findings of previous studies (Sanchez-Vives and McCormick, 2000, Seamans et al., 2003). There are several differences between the studies that could explain this apparent discrepancy. First, different species were used. The former studies used ferrets and rats respectively, as opposed to mice used in our experiments. Second, the former studies used non-NMDA receptor antagonists such as CNQX and 2,3-dihydroxy-6-nitro-7-sulfamoyl-benzo[f]quinoxaline-2,3-dione (NBQX), which also block kainate receptors, precluding their main use in our study; however, our trials on evoked Up states in the BC with CNQX confirmed the previously reported finding that Up states can still be evoked when AMPA receptors are blocked more completely (Favero and Castro-Alamancos, 2013). While it seems that NMDA receptor activation is necessary to maintain Up state activity in the barrel cortex, our stimulating pulse to evoke the Up state likely generated sufficient direct depolarisation to allow such NMDA receptor activation, due to the placement of the stimulating electrode in the local network. Therefore, in these experiments we were able to dissociate information regarding Up state maintenance from initiation.

### 4.7 Presynaptic vs. postsynaptic effects

The role of presynaptic receptors should also be considered. NMDA receptor blockade in the mEC reduced spontaneous Up state incidence, and made Up states less likely to be evoked by electrical stimulation. The mechanism for this is not immediately clear; while it could represent a simple reduction in overall network excitability due to the removal of an excitatory postsynaptic current, it is also possible that the effect of blockade of presynaptic NMDA receptors could be involved. Presynaptic NMDA receptor activation enhances neurotransmitter release (Banerjee et al., 2016). Therefore, antagonism at these receptors may lead to a reduction in neurotransmitter release, as compared to that in control conditions; this could conceivably reduce Up state incidence, essentially by further reducing network excitation.

In our current clamp experiments, UBP-302 application brought about a significant reduction in Up state duration in the mEC. However, the non-NMDA receptor mediated slow component was not removed entirely by UBP-302 in our voltage clamp experiments. There are two main possibilities that explain this potential discrepancy. Firstly, it could be the case that UBP-302 reduces the postsynaptic kainate current below a threshold at which it can no longer sustain prolonged Up state activity. A second possibility is that UBP-302 could also be having an effect at kainate receptors located elsewhere, namely at presynaptic locations or on interneurons. Presynaptic kainate receptors in the entorhinal cortex have been shown to be sensitive to UBP-302 (Chamberlain et al., 2012). It therefore could be the case that the observed effect on Up state duration arises from either or both of presynaptic and postsynaptic effects. However, our computational model has demonstrated the sufficiency of a postsynaptic kainate receptor-mediated current in maintaining the Up state.

### 4.8 Age of animals used in this study

In this study, slices were all obtained from mice aged between 14 and 19 postnatal days. This range was kept limited in order to mitigate the effects of any changes in receptor expression or network structure during development. However, it is important to consider that glutamate receptors and their subunits are expressed differently during the course of development (Erdö and Wolff, 1990, Feldmeyer and Cull-Candy, 1996, Franciosi, 2001). Therefore, one cannot rule out the possibility that our results are relevant primarily for young animals. However, it seems likely that these results hold relevance for older animals as well, due to the fact that general trends in Up state properties in control conditions are in keeping with those observed *in vivo* by Hahn et al. (2012), who utilised an older, broader age range of P26–42.

### 4.9 Implications

The mEC acts as an interface between the neocortex and the hippocampus, a role highly conserved across mammalian species (Van Strien et al., 2009, Insausti et al., 1987, Insausti, 1993). Therefore, there are network implications of a prolonged Up state in the mEC, as it would decouple the neocortex from the entorhinal cortex-hippocampal circuit activity (Hahn et al., 2012). As neocortico-hippocampal interaction is believed to be important for memory processing (Buzsáki, 1996), this information gating provided by the mEC may be computationally utilised for consolidation of different types of memory (Dupret and Csicsvari, 2012). Our findings suggest that kainate receptors may have a fundamental role in this gating function.

However, these properties of kainate receptors may also leave the region vulnerable to hyperexcitability. A relationship between seizure activity and Up state activity has been proposed (Žiburkus et al., 2013), and the entorhinal cortex is a prime focus for spontaneous epileptic activity (Vismer et al., 2015). Layer III pyramidal neurons of the mEC have been shown to degenerate selectively in kainate-induced animal models of epilepsy (Sperk et al., 1983), and in tissue samples from human patients extracted during surgical treatment of epilepsy (Du et al., 1993). Thus, it is possible that the kainate receptors that give this region its apparently unique Up state properties are the same that bestow such epileptic susceptibility upon it.

## Figures and Tables

**Table 1:** Parameters of integrate-and-fire neurons.

	Δ*V* (mV)	*V*_*Thres*_ (mV)	*θ*_*Thres*_ (mV)	*C* (pF)	*g*_*L*_ (nS)	*V*_*reset*_ (mV)	*V*_*L*_ (mV)	*g*_*Ad*_ (nS)	*V*_*Ad*_ (mV)	*tau*_*Ad*_ (ms)
E cells	2.5	−50	0	20	2	[−72, −68]	−70	7.5	−85	100
I cells	2.5	−50	0	200	10	[−82, −78]	−70	0	–	–

**Table 2:** Synaptic parameters used in the model of integrate-and-fire neurons.

Currents	*g* (nS)	E_*rev*_ (mV)	tau (ms)
AMPA_E-E_	35	0	2^a^
AMPA_E-I_	5.5	0	2^a^
KA_E-E_	245	0	100
GABA_A I-E_	72	−75^b^	5

a Compte et al. (2003).

b Bartos et al. (2001).

**Table 3:** Compiled attributes of Up and Down states across all recorded cortical regions. Data are presented as mean ± SEM.

	mEC (n = 31)	BC (n = 30)	BC LIII (n = 15)	BC LV (n = 15)
Up State Duration (s)	4.24 ± 0.28	2.60 ± 0.22	2.97 ± 0.25	2.21 ± 0.34
Up State Incidence (Hz)	0.047 ± 0.005	0.018 ± 0.002	0.015 ± 0.001	0.021 ± 0.003
Down State Duration (s)	22.6 ± 3.6	59.1 ± 5.8	71.9 ± 10.6	49.6 ± 5.3
Up State V_m_ (mV)	−55.1 ± 3.4	−57.3 ± 0.6	−57.5 ± 1.0	−57 ± 0.8
Down State V_m_ (mV)	−65.1 ± 0.6	−64.5 ± 0.8	−66.1 ± 1.4	−62.8 ± 0.7
Spike Frequency (Hz)	3.33 ± 0.53	1.52 ± 0.22	1.29 ± 0.32	1.75 ± 0.31

**Table 4:** Up state duration and incidence recorded in mEC LIII, BC LIII and BC LV in control condition and after application UBP-302 or d-AP5 (n = 5 for all 3 different regions). Data are presented as mean ± SEM. *p < 0.05, **p < 0.01.

	mEC				BC LIII				BC LV			
	Ctrl	UBP	Ctrl	AP5	Ctrl	UBP	Ctrl	AP5	Ctrl	UBP	Ctrl	AP5
Up State Duration (s)	5.37 ± 0.93	1.99 ± 0.20*	3.56 ± 0.23	4.24 ± 0.48	3.88 ± 0.70	3.53 ± 0.34	2.92 ± 0.33	–	3.06 ± 0.89	3.32 ± 1.01	1.78 ± 0.18	–
Up State Incidence (Hz)	0.045 ± 0.011	0.016 ± 0.006*	0.069 ± 0.01	0.01 ± 0.004**	0.017 ± 0.004	0.014 ± 0.002	0.01 ± 0.002	0.0 ± 0.0**	0.022 ± 0.003	0.024 ± 0.005	0.03 ± 0.01	0.0 ± 0.0**

## References

[bib1] Bartos M., Vida I., Frotscher M., Geiger J.R., Jonas P. (2001). Rapid signaling at inhibitory synapses in a dentate gyrus interneuron network. J. Neurosci..

[bib2] Banerjee A., Larsen R.S., Philpot B.D., Paulsen O. (2016). Roles of presynaptic NMDA receptors in neurotransmission and plasticity. Trends Neurosci..

[bib3] Beltramo R., D'Urso G., Dal Maschio M., Farisello P., Bovetti S., Clovis Y., Lassi G., Tucci V., De Pietri Tonelli D., Fellin T. (2013). Layer-specific excitatory circuits differentially control recurrent network dynamics in the neocortex. Nat. Neurosci..

[bib4] Bettler B., Kaupmann K., Mosbacher J., Gassmann M. (2004). Molecular structure and physiological functions of GABA_B_ receptors. Physiol. Rev..

[bib5] Buzsáki G. (1996). The hippocampo-neocortical dialogue. Cereb. Cortex.

[bib6] Castro-Alamancos M.A., Favero M. (2015). NMDA receptors are the basis for persistent network activity in neocortex slices. J. Neurophysiol..

[bib7] Chamberlain S.E.L., Jane D.E., Jones R.S.G. (2012). Pre- and post-synaptic functions of kainate receptors at glutamate and GABA synapses in the rat entorhinal cortex. Hippocampus.

[bib8] Chauvette S., Volgushev M., Timofeev I. (2010). Origin of active states in local neocortical networks during slow sleep oscillation. Cereb. Cortex.

[bib9] Compte A. (2006). Computational and in vitro studies of persistent activity: edging towards cellular and synaptic mechanisms of working memory. Neuroscience.

[bib10] Compte A., Sanchez-Vives M.V., McCormick D.A., Wang X.-J. (2003). Cellular and network mechanisms of slow oscillatory activity (<1 Hz) and wave propagations in a cortical network model. J. Neurophysiol..

[bib11] Cossart R., Aronov D., Yuste R. (2003). Attractor dynamics of network UP states in the neocortex. Nature.

[bib12] Craig M.T., Mayne E.W., Bettler B., Paulsen O., McBain C.J. (2013). Distinct roles of GABA_B1a_- and GABA_B1b_-containing GABA_B_ receptors in spontaneous and evoked termination of persistent cortical activity. J. Physiol. Lond..

[bib13] Cui C., Mayer M.L. (1999). Heteromeric kainate receptors formed by the coassembly of GluR5, GluR6, and GluR7. J. Neurosci..

[bib14] Cunningham M.O., Pervouchine D.D., Racca C., Kopell N.J., Davies C.H., Jones R.S.G., Traub R.D., Whittington M.A. (2006). Neuronal metabolism governs cortical network response state. Proc. Natl. Acad. Sci. U. S. A..

[bib15] Dhillon A., Jones R.S. (2000). Laminar differences in recurrent excitatory transmission in the rat entorhinal cortex in vitro. Neuroscience.

[bib16] Du F., Whetsell W.O., Abou-Khalil B., Blumenkopf B., Lothman E.W., Schwarcz R. (1993). Preferential neuronal loss in layer III of the entorhinal cortex in patients with temporal lobe epilepsy. Epilepsy Res..

[bib17] Dupret D., Csicsvari J. (2012). The medial entorhinal cortex keeps up. Nat. Neurosci..

[bib18] Erdö S.L., Wolff J.R. (1990). Postnatal development of the excitatory amino acid system in visual cortex of the rat. Changes in ligand binding to NMDA, quisqualate and kainate receptors. Int. J. Dev. Neurosci..

[bib19] Favero M., Castro-Alamancos M.A. (2013). Synaptic cooperativity regulates persistent network activity in neocortex. J. Neurosci..

[bib20] Feldmeyer D., Cull-Candy S. (1996). Functional consequences of changes in NMDA receptor subunit expression during development. J. Neurocytol..

[bib21] Franciosi S. (2001). AMPA receptors: potential implications in development and disease. Cell Mol. Life Sci..

[bib22] Gerster W., Kistler W., Naud R., Paninski L. (2014). Neuronal Dynamics: from Single Neurons to Networks and Models of Cognition.

[bib23] Hahn T.T.G., McFarland J.M., Berberich S., Sakmann B., Mehta M.R. (2012). Spontaneous persistent activity in entorhinal cortex modulates cortico-hippocampal interaction in vivo. Nat. Neurosci..

[bib24] Hájos N., Ellender T.J., Zemankovics R., Mann E.O., Exley R., Cragg S.J., Freund T.F., Paulsen O. (2009). Maintaining network activity in submerged hippocampal slices: importance of oxygen supply. Eur. J. Neurosci..

[bib25] Insausti R. (1993). Comparative anatomy of the entorhinal cortex and hippocampus in mammals. Hippocampus.

[bib26] Insausti R., Amaral D.G., Cowan W.M. (1987). The entorhinal cortex of the monkey: II. Cortical afferents. J. Comp. Neurol..

[bib27] Kohl M.M., Shipton O.A., Deacon R.M., Rawlins J.N.P., Deisseroth K., Paulsen O. (2011). Hemisphere-specific optogenetic stimulation reveals left-right asymmetry of hippocampal plasticity. Nat. Neurosci..

[bib28] Major G., Tank D. (2004). Persistent neural activity: prevalence and mechanisms. Curr. Opin. Neurobiol..

[bib29] Mann E.O., Kohl M.M., Paulsen O. (2009). Distinct roles of GABA_A_ and GABA_B_ receptors in balancing and terminating persistent cortical activity. J. Neurosci..

[bib30] Neske G.T., Patrick S.L., Connors B.W. (2015). Contributions of diverse excitatory and inhibitory neurons to recurrent network activity in cerebral cortex. J. Neurosci..

[bib31] Oikonomou K.D., Short S.M., Rich M.T., Antic S.D. (2012). Extrasynaptic glutamate receptor activation as cellular bases for dynamic range compression in pyramidal neurons. Front. Physiol..

[bib32] Paternain A.V., Morales M., Lerma J. (1995). Selective antagonism of AMPA receptors unmasks kainate receptor-mediated responses in hippocampal neurons. Neuron.

[bib33] Rodríguez-Moreno A., González-Rueda A., Banerjee A., Upton A.L., Craig M.T., Paulsen O. (2013). Presynaptic self-depression at developing neocortical synapses. Neuron.

[bib34] Ruiz-Mejias M., Ciria-Suarez L., Mattia M., Sanchez-Vives M.V. (2011). Slow and fast rhythms generated in the cerebral cortex of the anesthetized mouse. J. Neurophysiol..

[bib35] Sanchez-Vives M.V., McCormick D.A. (2000). Cellular and network mechanisms of rhythmic recurrent activity in neocortex. Nat. Neurosci..

[bib36] Seamans J.K., Nogueira L., Lavin A. (2003). Synaptic basis of persistent activity in prefrontal cortex in vivo and in organotypic cultures. Cereb. Cortex.

[bib37] Sheroziya M., Timofeev I. (2014). Global intracellular slow-wave dynamics of the thalamocortical system. J. Neurosci..

[bib38] Shu Y., Hasenstaub A., McCormick D.A. (2003). Turning on and off recurrent balanced cortical activity. Nature.

[bib39] Sperk G., Lassmann H., Baran H., Kish S.J., Seitelberger F., Hornykiewicz O. (1983). Kainic acid induced seizures: neurochemical and histopathological changes. Neuroscience.

[bib40] Squire L.R. (1992). Memory and the hippocampus: a synthesis from findings with rats, monkeys, and humans. Psychol. Rev..

[bib41] Steriade M., McCormick D.A., Sejnowski T.J. (1993). Thalamocortical oscillations in the sleeping and aroused brain. Science.

[bib42] Steriade M., Nuñez A., Amzica F. (1993). A novel slow (< 1 Hz) oscillation of neocortical neurons in vivo: depolarising and hyperpolarising components. J. Neurosci..

[bib43] Stroh A., Adelsberger H., Groh A., Rühlmann C., Fischer S., Schierloh A., Deisseroth K., Konnerth A. (2013). Making waves: initiation and propagation of corticothalamic Ca^2+^ waves in vivo. Neuron.

[bib44] Tahvildari B., Wölfel M., Duque A., McCormick D.A. (2012). Selective functional interactions between excitatory and inhibitory cortical neurons and differential contribution to persistent activity of the slow oscillation. J. Neurosci..

[bib45] Van Strien N.M., Cappaert N.L.M., Witter M.P. (2009). The anatomy of memory: an interactive overview of the parahippocampal-hippocampal network. Nat. Rev. Neurosci..

[bib46] Vismer M.S., Forcelli P.A., Skopin M.D., Gale K., Koubeissi M.Z. (2015). The piriform, perirhinal, and entorhinal cortex in seizure generation. Front. Neural Circuits.

[bib47] West P.J., Dalpé-Charron A., Wilcox K.S. (2007). Differential contribution of kainate receptors to excitatory postsynaptic currents in superficial layer neurons of the rat medial entorhinal cortex. Neuroscience.

[bib49] Wester J.C., Contreras D. (2012). Columnar interactions determine horizontal propagation of recurrent network activity in neocortex. J. Neurosci.

[bib48] Žiburkus J., Cressman J.R., Schiff S.J. (2013). Seizures as imbalanced up states: excitatory and inhibitory conductances during seizure-like events. J. Neurophysiol..

